# Cellular Senescence Triggers Altered Circadian Clocks With a Prolonged Period and Delayed Phases

**DOI:** 10.3389/fnins.2021.638122

**Published:** 2021-01-25

**Authors:** Rezwana Ahmed, Yasukazu Nakahata, Kazuyuki Shinohara, Yasumasa Bessho

**Affiliations:** ^1^Laboratory of Gene Regulation Research, Division of Biological Science, Graduate School of Science and Technology, Nara Institute of Science and Technology (NAIST), Ikoma, Japan; ^2^Department of Neurobiology and Behavior, Nagasaki University Graduate School of Biomedical Sciences, Nagasaki, Japan; ^3^Department of Pharmaceutical Sciences, North South University, Dhaka, Bangladesh

**Keywords:** circadian, clock, senescence, aging, oxidative stress

## Abstract

Senescent cells, which show the permanent growth arrest in response to various forms of stress, accumulate in the body with the progression of age, and are associated with aging and age-associated diseases. Although the senescent cells are growth arrested, they still demonstrate high metabolic rate and altered gene expressions, indicating that senescent cells are still active. We recently showed that the circadian clock properties, namely phase and period of the cells, are altered with the establishment of replicative senescence. However, whether cellular senescence triggers the alteration of circadian clock properties in the cells is still unknown. In this study we show that the oxidative stress-induced premature senescence induces the alterations of the circadian clock, similar to the phenotypes of the replicative senescent cells. We found that the oxidative stress-induced premature senescent cells display the prolonged period and delayed phases. In addition, the magnitude of these changes intensified over time, indicating that cellular senescence changes the circadian clock properties. Our current results corroborate with our previous findings and further confirm that cellular senescence induces altered circadian clock properties, irrespective of the replicative senescence or the stress-induced premature senescence.

## Introduction

Cellular senescence is the state of permanent growth arrest of cells. The senescent cells have been found to be accumulated in the body with aging, and have been associated with various age-related diseases, for example, atherosclerosis (Wang and Bennett, 2012; Childs et al., 2018; Cho et al., 2020), osteoarthritis (Jeon et al., 2017, 2019; Xu et al., 2017), alveolar lung diseases (Hashimoto et al., 2016; Schafer et al., 2017; Houssaini et al., 2018), and cancer (Parrinello et al., 2005; Bavik et al., 2006; Liu and Hornsby, 2007; Bhatia et al., 2008; Campisi et al., 2011; Castro-Vega et al., 2015; Ortiz-Montero et al., 2017). Removal of the senescent cells from the body, either using the pharmacologic interventions (Chang et al., 2016; Yosef et al., 2016; Baar et al., 2017; Lehmann et al., 2017; Schafer et al., 2017; Bussian et al., 2018; Zhang et al., 2019) or genetic ablations (Baker et al., 2011, 2016; Childs et al., 2016; Hashimoto et al., 2016; Bussian et al., 2018), have recently been reported to lead to the extended healthspan of prematurely and naturally aged mice and also attenuated the already existing diseases in mouse models of disease. Various forms of stress such as excessive cell proliferation, oncogenic stress and extreme DNA damage induce cellular senescence. These different forms of stress lead to the cells having the different types of the cellular senescence, such as the replicative senescence, oncogene-induced senescence and the stress-induced premature senescence. Despite the fact that the various types of the senescent cells are permanently growth arrested, they still have their individual differential transcriptome signatures, and secretory phenotype (Maciel-Baron et al., 2016; Hernandez-Segura et al., 2017; Nakao et al., 2020). Hence it can be postulated that the presence of the replicative senescent cells, oncogene-induced senescent cells, and stress-induced premature senescent cells may affect the physiological systems differentially. *In vivo* it is currently impossible to distinguish between the different types of the senescent cells and the effects they exert (Hernandez-Segura et al., 2017). Recently, we found that circadian clock properties are altered with replicative senescence. However, whether the alteration of the circadian clock is specific for the replicative senescent cells or is also observed in the other types of senescence programs is still largely known.

The circadian clock, which is an intrinsic time-keeping system of almost all living systems on earth, possesses robust and flexible mechanisms against environmental light/dark condition (Partch et al., 2014; Bass and Lazar, 2016; Takahashi, 2017; Honma, 2018). However, it has been found that the circadian clock becomes less robust and flexible with aging, both at the animal level (Valentinuzzi et al., 1997) and also at the tissue levels (Nakamura et al., 2015). Also, at the cellular level, we recently found that the circadian clock is altered with the establishment of replicative senescence; the circadian period becomes longer, and the peak phases are delayed compared with the proliferative cells (Ahmed et al., 2019). We assume that cellular senescence affects the circadian clock mechanism, but not *vice versa*, since we have reported that the fibroblast cells derived from *Bmal1* knockout mice embryo in which circadian clock is completely disrupted, show the normal senescence process (Nakahata et al., 2018). Although in our previous paper, we showed that the circadian clock is altered with the establishment of replicative senescence, till date, no evidence has directly demonstrated that cellular senescence *per se* affects the circadian clock mechanism. Hence, in this study, we induce oxidative stress-induced premature senescence of human primary fibroblasts, to investigate whether other types of senescence affect the circadian clock and therefore, confirm that cellular senescence affects the circadian clock, irrespective of the type of senescence.

## Materials and Methods

### Cell Culture and H_2_O_2_ Treatment

Primary human lung fetal fibroblasts (TIG-3) of Japanese origin were kindly provided by Drs. T. Takumi and T. Akagi. The cells were cultured in DMEM-4.5 g/L glucose (Nacalai Tesque, Japan) supplemented with 10% FBS (Sigma) and antibiotics (100 units/mL penicillin, 100 μg/mL streptomycin, Nacalai Tesque, Japan) at 37°C and 5% CO_2_ in a humidified incubator. The proliferative cells used in this study were established in our previous report (Ahmed et al., 2019) which consisted of cells in the passage range of P25-29.

**FIGURE 1:**
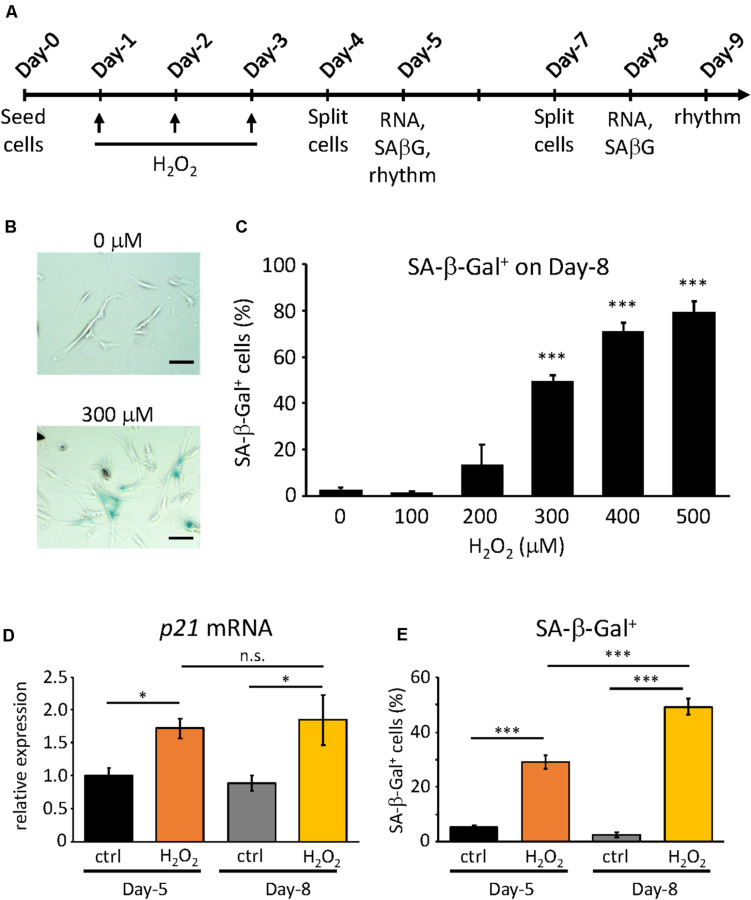
Confirmation of H_2_O_2_-induced premature senescence. **(A)** Scheme of this study. RNA, SA-β-Gal and rhythm mean RNA extraction, SA-β-Gal assay and real-time luciferase monitoring assay, respectively **(B)** Representative pictures of SA-β-Gal positive cells (blue) at Day-9 under control (upper) and 300 μM H_2_O_2_-treated conditions. Scale bars represent 100 μm (micrometer). **(C)** The percentage of SA-β-Gal positive cells were quantified at different concentrations. **(D)** *p21^*CIP*1^* gene expressions under control or 300 μM H_2_O_2_-treated conditions at indicated days were analyzed by qPCR. The expression level of control condition at Day-5 was set to 1. **(E)** The percentage of SA-β-Gal positive cells under control or 300 μM H_2_O_2_-treated conditions at indicated days were quantified. n.s., not significant, ^∗^*p* < 0.05, ^∗∗∗^*p* < 0.001, by Student’s two-tailed *t* test.

For the induction of oxidative stress-induced premature senescence H_2_O_2_ was used as the stressor. The proliferative cells were plated on 6-well plates at the seeding density of 8.5 × 10^4^ cells/well on Day-0 (Figure 1A). On Day-1, (i.e., 24 h after plating) cells were incubated with various concentrations of H_2_O_2_ (Wako, Japan) as indicated, for 2 h, then rinsed with DMEM twice and incubated for 22 h. Cells treated with equivalent volumes of dH_2_O as H_2_O_2_ were considered as controls. This process was repeated on Day-2 and Day-3. Then cells were cultured until Day-9, splitting on Day-4 and Day-7, each time with the seeding density at 8.5 × 10^4^ cells/well.

### Lentivirus Production and TIG-3 Cells Infection

Lentivirus production was performed as described previously (Ahmed et al., 2019). For infection of the target TIG-3 cells, cells in the Passage range of 25–29 in the previous study (Ahmed et al., 2019) were used. The culture medium was replaced with the lentivirus suspension supplemented with 8 μg/ml protamine sulfate (Nacalai Tesque, Japan). 24 h later the cells were washed with PBS once and cultured 2 more days with fresh medium. Infected cells were kept in liquid nitrogen until cells were subjected to experiments.

### Senescence-Associated β-Galactosidase Assay (SA-β-Gal Assay), RNA Extraction, qPCR, Real-Time Luciferase Monitoring Assay, and Cosinor Analysis

These methods were described preciously (Ahmed et al., 2019).

### Statistics

Values are reported as mean ± SEM. Statistical differences were determined by a Student’s two-tailed *t* test. Statistical significance is displayed as ^∗^*p* < 0.05, ^∗∗^*p* < 0.01, or ^∗∗∗^*p* < 0.001.

## Results

### Oxidative Stress-Induced Premature Senescence in TIG-3 Cells

In our previous study, we obtained the proliferative and replicative senescent TIG-3 cells by serial passaging and found that senescent TIG-3 cells possess altered circadian clock properties with prolonged period and delayed phase (Ahmed et al., 2019). To address whether the senescence process triggers the alteration of circadian clock properties, we induced the oxidative stress-induced premature senescence using the proliferative cells, which consisted of cells in the Passage range of 25–29 in the previous study (Ahmed et al., 2019). In order to induce oxidative stress-induced premature senescence, we chose H_2_O_2_, as it is one of the most widely used stressors and also because it is thought of as a natural inducer of oxidative stress (Toussaint et al., 2000). To optimize the concentration of H_2_O_2_, we first exposed the cells to varying concentrations of H_2_O_2_ for 2 h, performing 3 consecutive H_2_O_2_ treatments every 24 h (Figure 1A). Senescent cells are known to exhibit a plethora of features such as enlarged, flattened morphology, increased senescence-associated β-galactosidase (SA-β-Gal) activity (Debacq-Chainiaux et al., 2009; Khaidizar et al., 2017), and increased expressions of cell cycle inhibitors (*p16^*INK*4*a*^, p19^*ARF*^*, and *p21*^*CIP*1^) (Stein et al., 1999; Krishnamurthy et al., 2004; Khaidizar et al., 2017). Hence on Day-8, the cells were checked for some of the aforementioned features. Starting at 300 μM, the cells appeared to be larger in size and flattened and showed significantly higher percentage of the SA-β-Gal-positive cells compared to the control cells (Figures 1B,C). Higher concentrations also gave correspondingly higher percentage of SA-β-Gal-positive cells, however, increasing number of cell deaths also occurred. As such, we determined that the optimum concentration that could induce significant SA-β-Gal activity was 300 μM (49.3% ± 2.9), and this percentage of SA-β-Gal-positive cells was in the same range to that found in the replicative senescent cells, as reported previously (Ahmed et al., 2019).

Next, we sought to characterize the process of the oxidative stress-induced premature senescence after the exposure of TIG-3 cells to H_2_O_2_. To this end, we checked the two senescence features, the cell cycle inhibitor *p21* mRNA expression level and SA-β-Gal activity, at two time points i.e., on Day-5 and Day-8 at 300 μM of H_2_O_2_. On Day-5, both *p21* mRNA expression (*p* = 0.02) and SA-β-Gal positive cells (*p* = 4.8 × 10^–8^) in the H_2_O_2_-treated cells were increased compared to the control cells (Figures 1D,E). As expected, on Day-8 both senescence features were significantly higher in H_2_O_2_-treated cells (*p* = 0.01 and 5.5 × 10^–5^ for *p21* mRNA expression and SA-β-Gal positive cells, respectively). Intriguingly, *p21* expression levels were comparable in H_2_O_2_-treated cells between Day-5 and Day-8 (*p* = 0.77, Figure 1D), whereas SA-β-Gal positive cells on Day-8 was significantly higher than that on Day-5 (*p* = 1.6 × 10^–12^, Figure 1E). These results indicate that the cells start ceasing proliferation almost immediately after exposure to the stressor H_2_O_2_, however, the development of the oxidative stress-induced premature senescence process is gradual, the intensity of which increases with time.

### Alteration of Circadian Clock Characteristics in the Oxidative Stress-Induced Premature Senescent TIG-3 Cells

**FIGURE 2:**
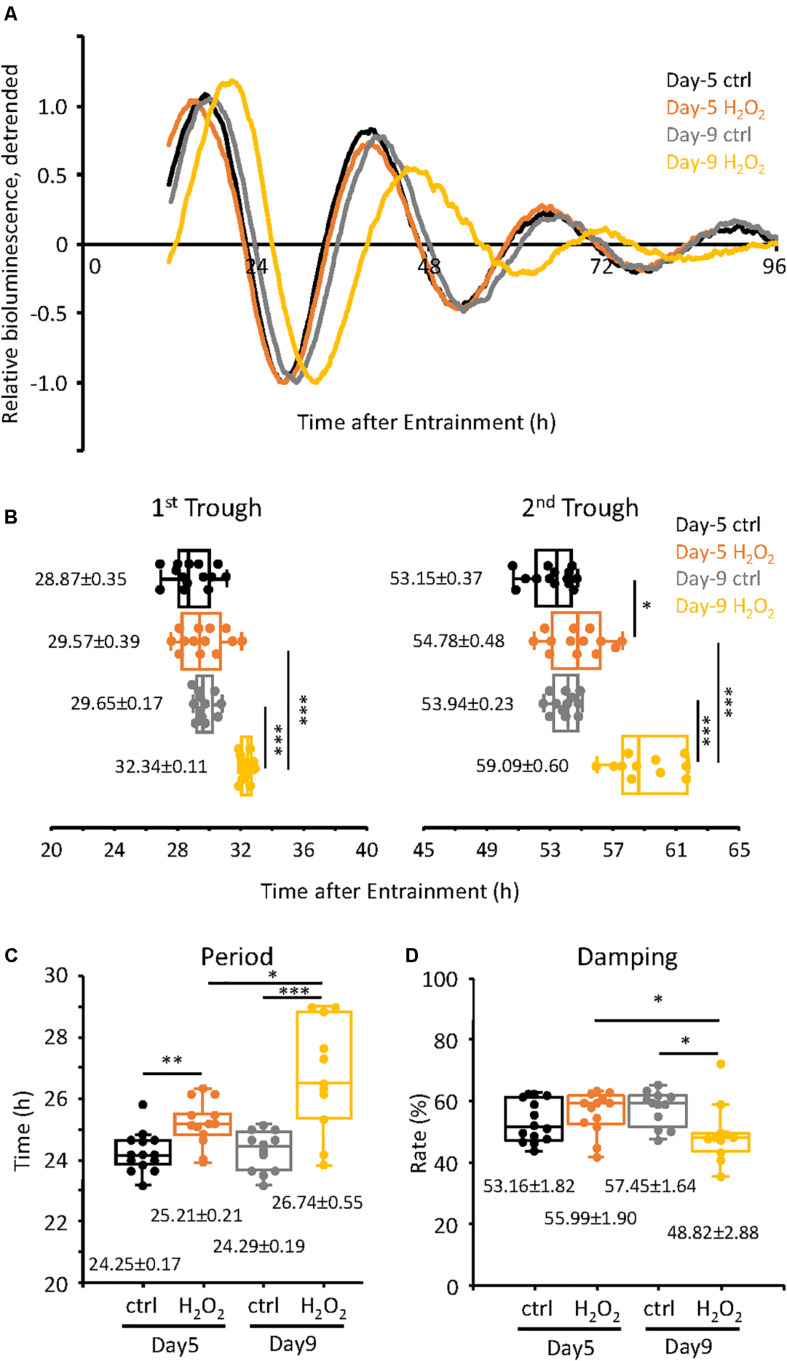
Alteration of circadian clock in H_2_O_2_-induced premature senescent cells at Day-9 was observed. **(A)** Relative oscillation patterns of luciferase of control and 300 μM H_2_O_2_-treated cells at Day-5 and -9 were monitored by using a real-time luciferase monitoring system. Lowest intensity of each sample was set to –1. **(B)** Box-whisker plots of trough-times are displayed. Values are mean ± SEM. **(C,D)** Box-whisker plots of period lengths **(C)** and damping ratio **(D)** in cells with control and H_2_O_2_-treated cells at Day-5 and -9 are displayed. Values are mean ± SEM. ^∗^*p* < 0.05, ^∗∗^*p* < 0.01, and ^∗∗∗^*p* < 0.001, by Student’s two-tailed *t* test.

Next we assessed the changes in the circadian clock properties of the cells, both at Day-5 and Day-9, compared to the control cells. For this purpose, we used the TIG-3 cells lentivirally infected with the *bmal1* promoter-driven luciferase gene (Brown et al., 2005). The infected cells were synchronized with dexamethasone and were subjected to real-time luciferase assay. As shown in Figure 2A, the circadian oscillation patterns of the control cells both on Day-5 and Day-9 were very close to each other (see Supplementary Figure 1 for raw data of oscillation patterns). Intriguingly, the oscillation pattern of H_2_O_2_-treated cells on Day-5, which have already shown senescent features (Figures 1D,E), was similar to those of control cells, suggesting that circadian clock is intact in Day-5 senescent cells. On the contrary, the oscillation pattern of the H_2_O_2_-treated cells on Day-9 was shifted to the right (Figure 2A), suggesting the alteration of the circadian clock i.e., a delay in their clock timings. In order to more precisely check the timings of the cells, the trough times of the cells on Day-5 and Day-9 were extracted. For the cells on Day-5, the 1st trough times were 28.87 ± 0.35 h and 29.57 ± 0.39 h for the control cells and H_2_O_2_-treated cells, respectively, with no statistically significant difference (*p* = 0.19, Figure 2B). For the cells on Day-9, the 1st trough times were 29.65 ± 0.17 h and 32.34 ± 0.11 h for the control cells and H_2_O_2_-treated cells, respectively, with statistically significant difference (*p* = 1.5 × 10^–11^). For the 2nd trough times, the control cells at Day-5 showed 53.15 ± 0.37 h while the H_2_O_2_-treated cells showed 54.78 ± 0.48 h, with statistically significant difference (*p* = 0.01). For the Day-9 cells, the 2nd trough times were 53.94 ± 0.23 h and 59.09 ± 0.60 h, for the control cells and H_2_O_2_-treated cells, respectively, with statistically significant difference (*p* = 4.4 × 10^–8^). We then compared intra-group trough times of the cells between Day-5 and Day-9. As expected, there were no differences of 1st and 2nd trough times in the control cells. On the other hand, for 1st trough times in H_2_O_2_-treated cells, the cells at Day-5 showed 29.57 ± 0.39 h while the cells at Day-9 showed 32.34 ± 0.11 h, with statistically significant difference (*p* = 1.9 × 10^–6^). Also, for 2nd trough times in H_2_O_2_-treated cells, the cells at Day-5 showed 54.78 ± 0.48 h and the cells at Day-9 showed 59.09 ± 0.60 h, with statistically significant difference (*p* = 1.0 × 10^–5^). These results indicate that the H_2_O_2_-treated cells on Day-9, with the higher level of the senescent features, consistently displayed the delayed trough timings, which is in accordance with the replicative senescent cells reported in our previous study (Ahmed et al., 2019). Meanwhile, the trough timings of H_2_O_2_-treated cells on Day-5, with the milder level of the senescent features, were similar to those of control cells, which suggests that the alteration of circadian clock by H_2_O_2_ on Day-5 is much milder than that on Day-9.

We further checked the period and damping rate of the cells on Day-5 and Day-9 (Figures 2C,D). Period was calculated as time difference between 1st and 2nd trough times. For the cells on Day-5, the period length of the control cells was 24.25 ± 0.17 h and that of the H_2_O_2_-treated cells was 25.21 ± 0.21 h, *p* = 0.002, with a period extension of 0.96 h in the H_2_O_2_-treated cells. For the cells at Day-9, the period of the control cells was 24.29 ± 0.19 h while that of the H_2_O_2_-treated cells was 26.74 ± 0.55 h, *p* = 2.8 × 10^–4^, with a period extension of 2.45 h. Furthermore, the period of the H_2_O_2_-treated cells on Day-9 was 1.53 h longer than that on Day-5, with statistically significant different (*p* = 0.011). In case of the damping rate of the circadian oscillation patterns of the cells, Day-5 cells did not show any significant difference in their damping rates, for both the control and H_2_O_2_-treated cells (Figure 2D). For the cells of Day-9, the oscillation pattern of the H_2_O_2_-treated cells damped down more than the control cells, *p* = 0.015. Also, the damping of the H_2_O_2_-treated cells on Day-9 damped down more than that on Day-5, *p* = 0.044. Collectively, the period changes and damping rates suggest that the H_2_O_2_-treated cells on Day-9 display the higher intensity alterations of the circadian clock properties, although period changes start with the initiation of the process of oxidative stress-induced premature senescence.

To confirm the above results, we also analyzed the data of Figure 2 mathematically using the Cosinor software (Supplementary Figure 2). For the period, control cells on Day-5 had the period of 23.70 ± 0.21 h while the H_2_O_2_-treated cells had period of 25.99 ± 0.14 h, *p* = 2.6 × 10^–9^; for Day-9 cells, the period of the control cells was 25.03 ± 0.16 h while that of the H_2_O_2_-treated cells was 26.85 ± 0.19 h, *p* = 2.5 × 10^–7^ (Supplementary Figure 2A), both of which are consistent with the manual extraction of the period data (Figure 2). Again, the period of H_2_O_2_-treated cells on Day-9 was significantly longer than that on Day-5, *p* = 0.001. In case of the acrophase, on Day-5, the control cells had an acrophase of −320.00 ± 5.76 while the H_2_O_2_-treated cells had an acrophase of −317.69 ± 5.00, with no statistically significant difference (*p* = 0.77, Supplementary Figure 2B), indicating that there is no phase delay between the cells at the beginning of the oxidative senescence development. On Day-9, the control cells had an acrophase of −322.25 ± 2.36, which was comparable to those of Day-5, while the H_2_O_2_-treated cells had an acrophase of −356.64 ± 1.06, *p* = 2.0 × 10^–11^ (Supplementary Figure 2B), indicating phase delay on Day-9 in accordance with Figure 2B. Also, the acrophase of H_2_O_2_-treated cells on Day-9 was significantly different from that on Day-5, *p* = 0.001.

3xH_2_O_2_ treatment induced the initiation of cellular senescence easily, however, it altered all the circadian clock properties gradually. Therefore, we conclude that the circadian changes observed in the H_2_O_2_-treated cells are a result of the oxidative stress-induced premature senescence of the cells, not simply an effect of the H_2_O_2_ on the cells *per se*.

**FIGURE 3:**
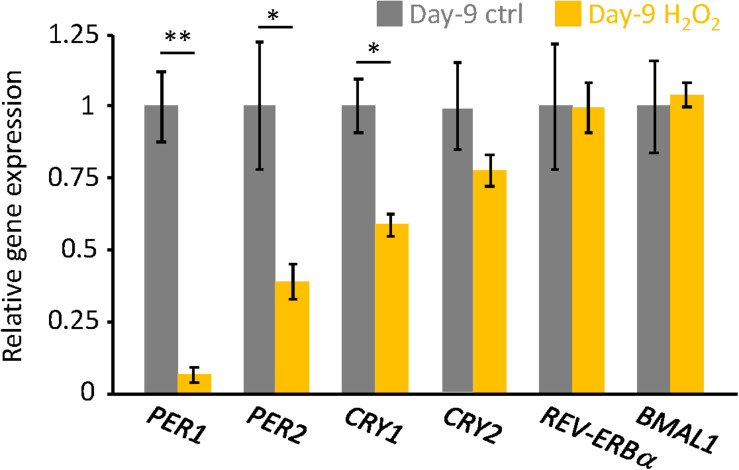
The endogenous circadian clock genes expression level was downregulated in the senescent cells. *PER1*, *PER2*, *CRY1*, *CRY2*, *REV-ERBa*, and *BMAL1* mRNAs in unsynchronized cells were analyzed by qPCR. Each sample was normalized by 18S rRNA. Expression levels of each gene in control cells were set to 1. ^∗^*p* < 0.05, ^∗∗∗^*p* < 0.01, by Student’s two-tailed *t* test.

Finally, endogenous circadian gene expressions in H_2_O_2_-induced senescent cells on Day-9 were analyzed. Similar to our previous results in replicative senescent cells (Ahmed et al., 2019), *PER1*, *PER2*, and *CRY1* mRNAs were downregulated in senescent cells, however, *CRY2*, *REV-ERBa*, and *BMAL1* mRNAs were comparable to control non-senescent cells (Figure 3). These results suggest that not only circadian phenotypes, but also molecular regulations for circadian clock are similar, irrespective of the type of cellular senescence.

## Discussion

In this study, we revealed that the oxidative stress-induced premature senescence triggers the alteration of circadian clock properties, that is, the delayed phase and period extension. Also, we have recently reported that the period and phase of circadian clock in the replicative senescent cells was prolonged and delayed compared to the proliferative cells, respectively (Ahmed et al., 2019). Based on our findings, we propose that cellular senescence induces the period extension and delayed phase of circadian clock properties by similar molecular mechanisms, irrespective of the replicative senescence or the oxidative stress-induced premature senescence.

In aged organisms, in addition to the replicative senescent cells, the stress-induced premature senescent cells occupy a major portion of the senescent cells (Campisi, 2005; Khapre et al., 2011). Oxidative stress is one of the strongest contributors of stress-induced premature senescence and is likely one of the major mediators of stress-induced premature senescence *in vivo* (Chen et al., 1995; Campisi, 2005; Khapre et al., 2011). Interestingly, several studies from model animals and humans have demonstrated that aging can also lead to alteration of the circadian clock (Pittendrigh and Daan, 1974; Witting et al., 1994; Valentinuzzi et al., 1997; Van Someren, 2000; Davidson et al., 2008; Sellix et al., 2012; Mattis and Sehgal, 2016). These evidence suggest that the attenuation of circadian clock functions with aging is in accordance with the accumulation of the senescent cells *in vivo*. Senolytic drugs (Chang et al., 2016; Yosef et al., 2016; Lehmann et al., 2017) which selectively eliminate senescent cells, or transgenic mice, such as INK-ATTAC (Baker et al., 2011) and p16-3MR mice (Demaria et al., 2014), in which senescent cells can be selectively eliminated in an inducible fashion, will be good strategies to address this hypothesis.

As already discussed in our previous study (Ahmed et al., 2019), the altered circadian clock properties have also been reported by Nakamura et al. (2015) using *ex vivo* SCN tissue of old mice. Compared to the consistent results from cellular and tissue levels, results at the organismal level have been controversial, some reports demonstrate prolonged period (Valentinuzzi et al., 1997), but others show shortened period (Pittendrigh and Daan, 1974; Witting et al., 1994). Aging phenotype is the result of complex intra- and inter-organ communications and individual contributions of different factors to total aging phenotype are still unknown. This is probably the reason for the controversial reports at the organismal level. Further investigations to unravel individual factors affecting total aging phenotype will be required.

We concluded in this study that 3×H_2_O_2_-treated cells on Day-5 have already entered the senescent phase, because of the high expression and level of *p21* mRNA and SA-b-Gal activity, respectively (Figures 1D,E), and H_2_O_2_-treated cells on Day-9 were more maturated. Meanwhile the alteration of circadian clock properties in H_2_O_2_-treated cells on Day-5 occurred only in terms of the period prolongation, and on Day-9 the period was much longer than that on Day-5. Intriguingly, phase and damping rate were altered only on Day-9, suggesting that molecular mechanisms of the period prolongation and delayed-phase/damping are independent. These results also suggest that the molecular mechanisms in circadian period regulations are vulnerable to cellular senescence, while the molecular mechanisms in circadian phase regulations are more robust than those in period regulations. Compared to our 3xH_2_O_2_ treatment, acute single H_2_O_2_ treatment with high dose has been reported to alter circadian clock properties; H_2_O_2_ treatment resets circadian clock mediated by the dimerization of BMAL1 and HSF1 (Tamaru et al., 2013), induces phase changes of circadian clock in mouse embryonic fibroblast (MEF) cells and mouse peripheral tissues (Tahara et al., 2016), increases the amplitude of circadian clock by activating NRF2 following *Cry1* expression in stable Per2:Luc reporter MEF cells (Wible et al., 2018), elicits phase-dependent PER2 degradation and circadian phase shifts in mouse fibroblasts (Putker et al., 2018), and shortens the circadian period by downregulating *Rev-erva/b* mRNAs via the activation of PRX2/STAT3 pathway in stable Bmal1:dLuc reporter NIH3T3 cells (Ji et al., 2019). These circadian phenotypes triggered by acute H_2_O_2_ are different from our current results, thereby indicating that the circadian phenotypes observed in our study are a result of the oxidative stress-induced premature senescence of the cells, not simply an effect of the H_2_O_2_ on circadian clock *per se*.

Li et al. (2020) have recently reported that increased non-genetic variation in gene expression predominantly drives circadian period prolongation in clonal cell lines (Li et al., 2020). Our studies demonstrated that variations in trough times and periods are larger in replicative/stress-induced premature senescent cells, compared to those in proliferative/control cells. Meanwhile, senescent cells are not homogeneous, they are heterogenous mixture of cells, for example, the percentage of SA-b-Gal positive cells was not 100% (Figure 1D). These data support that variation in circadian gene expression among senescent cells is greater. Furthermore, aging has been associated with increased stochastic transcriptional noise (Bahar et al., 2006; Enge et al., 2017; Martinez-Jimenez et al., 2017; Tang et al., 2019), therefore, increased transcriptional noise in senescent cells might be one of the causes to induce prolonged circadian period. Analyses of circadian period in single cells and single-cell RNA-sequence will provide an answer for this possibility.

Senescent cells are metabolically active, and increase in the AMP/ATP ratio and decrease in NAD^+^ amount have been reported during senescence (James et al., 2015; Khaidizar et al., 2017). Increase in the AMP/ATP ratio promotes AMP-activated protein kinase (AMPK), which acts as a sensor of the reduced energetic state and further activates catabolic pathways while inhibiting anabolic ones (Hardie, 2003; Garcia and Shaw, 2017). Meanwhile it has been reported that mTOR, which is an intracellular nutrient sensor for high cellular energy state and associated with autophagy, is also upregulated during senescence (Herranz et al., 2015; Laberge et al., 2015; Nacarelli and Sell, 2017). Decrease in NAD^+^ amount attenuates enzymatic activities of NAD^+^-dependent enzymes, such as sirtuin family deacetylase (SIRT1-7) and poly (ADP-ribose) polymerases (PARPs) (Imai and Guarente, 2014, 2016; Schultz and Sinclair, 2016). Many of aforementioned signaling molecules are reported to regulate circadian clock properties. AMPK is a rhythmically expressed kinase and phosphorylates CK1ε, resulting in enhanced phosphorylation and degradation of PER2 (Um et al., 2007; Sahar and Sassone-Corsi, 2012) and CRY1 (Lamia et al., 2009; Sahar and Sassone-Corsi, 2012; Jordan and Lamia, 2013). AMPK activation by AMPK agonist, AICAR, or glucose deprivation, increased the circadian period and decreased the amplitude (Lamia et al., 2009), which are consistent with our finding in senescent cells, although another AMPK agonist metformin shortened circadian period (Um et al., 2007). mTOR perturbation, such as RNAi knockdown or mTOR inhibitors, lengthened circadian period in fibroblast, SCN, and animal behaviors (Zhang et al., 2009; Ramanathan et al., 2018), these reports show opposite effects to our findings. NAD^+^ shows rhythmic 24 h oscillation and post-translationally modifies histone H3, BMAL1, PER2 and CLOCK by SIRT1 and PARP1 (Nakahata et al., 2008; Ramsey et al., 2009; Asher et al., 2010). Decrease in NAD^+^ by FK866 treatment amplified E-box-regulated circadian genes, such as *per2* and *dbp* mRNAs (Nakahata et al., 2009). Current study demonstrated that the amplitude of circadian oscillation driven by *bmal1*-promoter was damped more in senescent cells (Figure 2D), which is probably due to the increase in E-box-regulated circadian gene, *rev-erb*, the repressor for *bmal1* gene regulation. Intriguingly, it has been demonstrated that H_2_O_2_ decreases intracellular NAD^+^ in some primary cells, suggesting that senescent cells in our study also possesses low NAD^+^ (Furukawa et al., 2007; Han et al., 2016). Evidences mentioned here imply that altered signaling pathways during senescence affects circadian clock properties, however, as far as we know, molecular connections between cellular senescence and circadian clock remain largely uncovered. Therefore, further investigations addressing this will be required to understand, maintain and cure the circadian clock mechanisms in the elderly.

In summary, our results indicate that cellular senescence alters the circadian clock, irrespective of the type of cellular senescence. In aged individuals, disruption of the circadian clock functions has been associated with many age-related diseases, however, the underlying cause of this disruption of the circadian clock was largely unknown. Our novel findings, therefore, open up new avenues to investigate the underlying mechanisms that lead to the disruption of the circadian clock function in aged organisms.

## Data Availability Statement

The original contributions presented in the study are included in the article/Supplementary Material, further inquiries can be directed to the corresponding author.

## Author Contributions

RA performed experiments and drafted the manuscript. YN performed experiments, designed the overall approach, coordinated the study, and wrote the manuscript. KS contributed to coordination of the study. YB contributed to design and coordination of the study. All authors contributed to the article and approved the submitted version.

## Conflict of Interest

The authors declare that the research was conducted in the absence of any commercial or financial relationships that could be construed as a potential conflict of interest.
