# Supplementary figures and images for: Cellular Senescence Triggers Altered Circadian Clocks With a Prolonged Period and Delayed Phases
Source: Front Neurosci. 2021 Jan 25;15:638122. doi: 10.3389/fnins.2021.638122 (PMC7868379; doi:10.3389/fnins.2021.638122)

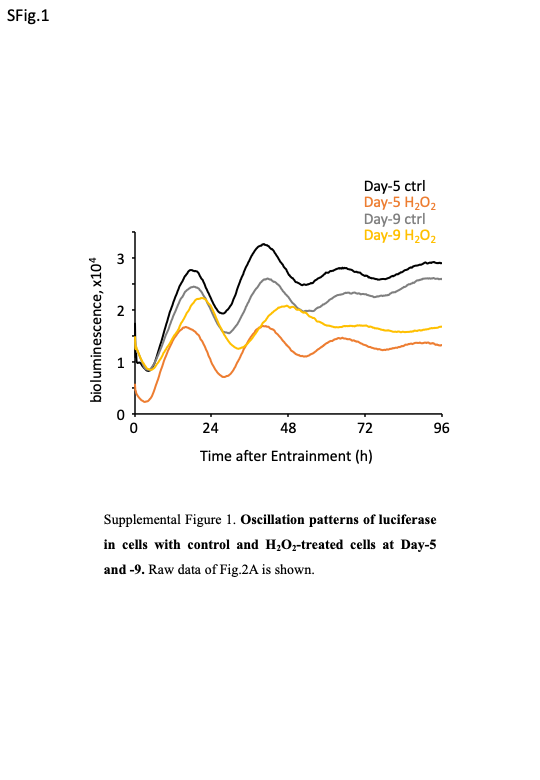

Supplement: Supplementary file 1 [file Image_1.tiff]

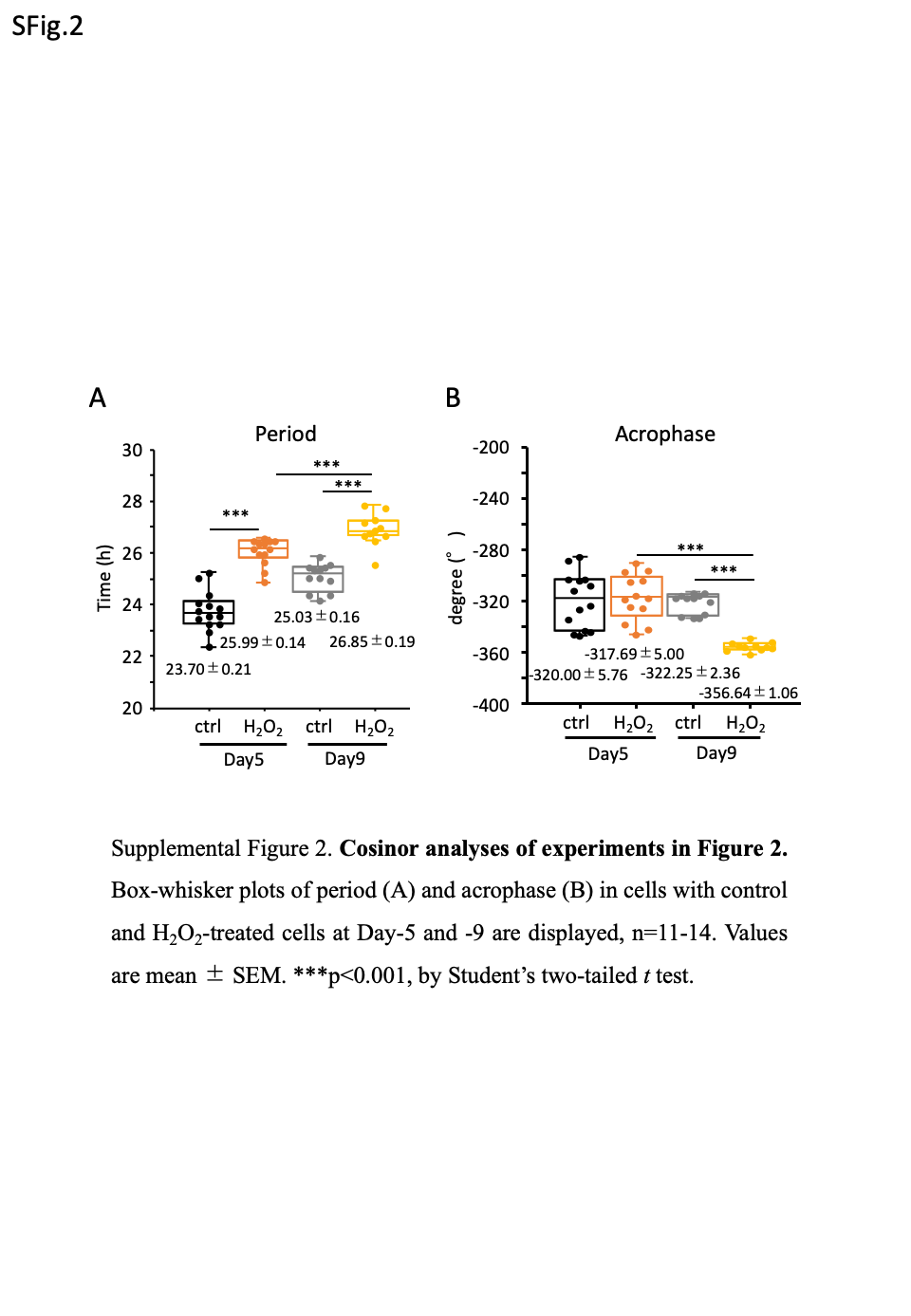

Supplement: Supplementary file 2 [file Image_2.tiff]
